# Analyses of In Vivo Interaction and Mobility of Two Spliceosomal Proteins Using FRAP and BiFC

**DOI:** 10.1371/journal.pone.0001953

**Published:** 2008-04-16

**Authors:** Gul Shad Ali, K. V. S. K. Prasad, M. Hanumappa, A. S. N. Reddy

**Affiliations:** Department of Biology and Program in Molecular Plant Biology, Colorado State University, Fort Collins, Colorado, United States of America; Umeå Plant Science Centre, Sweden

## Abstract

U1-70K, a U1 snRNP-specific protein, and serine/arginine-rich (SR) proteins are components of the spliceosome and play critical roles in both constitutive and alternative pre-mRNA splicing. However, the mobility properties of U1-70K, its in vivo interaction with SR proteins, and the mobility of the U1-70K-SR protein complex have not been studied in any system. Here, we studied the in vivo interaction of U1-70K with an SR protein (SR45) and the mobility of the U1-70K/SR protein complex using bimolecular fluorescence complementation (BiFC) and fluorescence recovery after photobleaching (FRAP). Our results show that U1-70K exchanges between speckles and the nucleoplasmic pool very rapidly and that this exchange is sensitive to ongoing transcription and phosphorylation. BiFC analyses showed that U1-70K and SR45 interacted primarily in speckles and that this interaction is mediated by the RS1 or RS2 domain of SR45. FRAP analyses showed considerably slower recovery of the SR45/U1-70K complex than either protein alone indicating that SR45/U1-70K complexes remain in the speckles for a longer duration. Furthermore, FRAP analyses with SR45/U1-70K complex in the presence of inhibitors of phosphorylation did not reveal any significant change compared to control cells, suggesting that the mobility of the complex is not affected by the status of protein phosphorylation. These results indicate that U1-70K, like SR splicing factors, moves rapidly in the nucleus ensuring its availability at various sites of splicing. Furthermore, although it appears that U1-70K moves by diffusion its mobility is regulated by phosphorylation and transcription.

## Introduction

Pre-mRNA splicing, the removal of introns and subsequent joining of exons in precursor mRNA is critical for the expression of most genes in eukaryotes. In addition, regulated alternative splicing, a process that produces multiple transcripts from the same gene, has been implicated in post-transcriptional gene regulation and in increasing proteome diversity, further reinforcing the importance of splicing in eukaryotes [1]–[5]). Splicing is a highly dynamic process that involves recruitment and massive rearrangement of five small nuclear ribonucleoprotein particles (snRNPs). Splicing is initiated by the binding of U1 snRNP on the 5′ splice site, committing pre-mRNA to splicing, which is followed by an orderly recruitment and dissociation of snRNPs ultimately resulting in the generation of mRNA [2], [6]. In metazoans, the recruitment of U1 snRNP to the 5′ splice site is facilitated by members of the serine/arginine-rich (SR) protein family [7]–[11]. These proteins, characterized by the presence of RNA binding domains and a serine-arginine dipeptide rich region, the RS domain, interact with U1 snRNP and pre-mRNA simultaneously and, hence, promote the correct identification of the 5′ splice site [8], [12]. In multicellular eukaryotes, sequences around the splice sites are less conserved, and, therefore, for correct and efficient definition of splice sites, additional regulatory sequences adjacent to the splice sites, collectively called splicing enhancers/repressors, are required [1], [4], [12]. SR proteins are thought to bind to these sequences and promote the recruitment of U1 snRNP to the correct 5′ splice sites [7]–[11]. In addition, SR proteins also modulate the selection of alternative weak splice sites and are probably the major contributors to increasing the transcriptome complexity and protein diversity [8].

U1 snRNP consists of U1 sn RNA and several proteins [13], [14]. These proteins are required for the assembly of U1 snRNP on the splice sites, primarily by facilitating base pairing between U1 snRNA and the nucleotides around the 5′ exon/intron junction. The Arabidopsis U1-70K, one of the U1 snRNP-specific proteins, is structurally similar to the U1-70Ks from metazoans [15]. It has an RNA recognition motif (RRM), which is involved in binding to U1 snRNA, and an arginine-rich C-terminal region. In addition, it has been shown to interact with mammalian SR splicing factors (ASF/SF2 and SC35) and it is suggested that SR proteins function in recruitment of U1 snRNP to 5′ splice sites by this interaction [16]–[18]. The Arabidopsis U1-70K also interacts with several SR proteins [1], [19]–[21] implying that U1-70K plays an important role in pre-mRNA splicing in diverse organisms. Given the critical role of U1-70K in splicing, it is essential to address several cell biological questions related to the function of U1-70K. For example, it is not known if U1-70K is statically bound to its targets or is in a continuous steady-state flux between various subnuclear domains. Since U1-70K plays an important role in splicing we asked whether its subcellular distribution and kinetics are altered by inhibition of transcription. Also, U1-70K is heavily phosphorylated, and phosphorylation and dephosphorylation play important roles in the assembly and progression of splicing [15], [22], [23]. These observations prompted us to study the regulation of subcellular distribution and mobility of U1-70K by phosphorylation. Here, we first assessed these biophysical properties of U1-70K in living cells by FRAP analyses with GFP-tagged U1-70K protein expressed in Arabidopsis cells under control conditions or when treated with inhibitors of transcription, and protein phosphorylation and dephosphorylation. With FRAP analyses, we show that inhibition of transcription and protein phosphorylation and dephosphorylation slowed its mobility, indicating that the mobility of U1-70K is regulated by transcription and the phosphorylation status of the cell.

We have recently shown that SR45, one of the plant-specific SR proteins, plays a role in constitutive and alternative splicing and affects multiple developmental processes [24]. Previously, using yeast two hybrid and pull-down assays, we showed that SR45 interacts with U1-70K [21], [25]. These studies, however, did not reveal the spatial and temporal interaction of SR45 and U1-70K in living plant cells. To address these issues, we used a bimolecular fluorescence complementation (BiFC) system, which allows analysis of in vivo protein-protein interactions [26]. Using the same BiFC system together with deletion mutants consisting of various combination of RS1, RNA recognition motif (RRM) and RS2 of SR45, we mapped domains in SR45 that interact with U1-70K. To understand if the subnuclear organization of the SR45/U1-70K complex is affected by the phosphorylation and transcription status of the cell, we examined the cells for a spatial re-organization of SR45/U1-70K BiFC complexes after treatment with inhibitors of phosphorylation and transcription. Since BiFC results from the reconstitution of YFP fluorescence through the interactions of their tagged proteins, it provided us with an opportunity to study the dynamics of the U1-70K/SR45 complex by FRAP analysis.

## Results

### Mobility of U1-70K is affected by the status of transcription and protein phosphorylation

**Figure 1:**
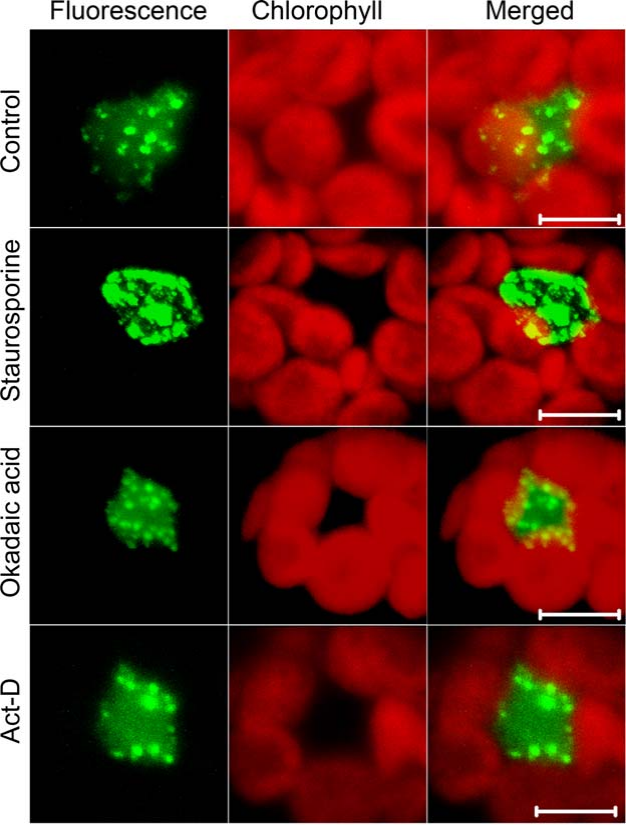
Subnuclear distribution of U1-70K is affected by protein phosphorylation but not by transcription and dephosphorylation. Confocal images of Arabidopsis protoplasts expressing GFP-U1-70K that were either left untreated (control) or treated with staurosporine, okadaic or actinomycin-D (Act-D) for 2 hours. Inhibition of phosphorylation redistributes U1-70K to larger speckles (Staurosporine). Shown are representative images presented as projections of 10 z-stack slices each 0.5 µm apart of at least 50 examined protoplasts. Bar = 5 µm.

Several spliceosomal proteins are known to distribute in a characteristic pattern of speckles in the diffuse background of nucleoplasm [27], [28]. The GFP-tagged U1-70K also showed a similar fluorescence pattern in the Arabidopsis mesophyll protoplasts with brighter foci and a substantial nucleoplasmic distribution (Figure 1). Currently there is no U1-70K mutant, which could be used for complementation with fluorescent protein tagged U1-70K fusion constructs. However, the GFP-U1-70K and the RFP-U1-70K fusions under normal conditions displayed a localization pattern that was expected, i.e. in speckles and in the nucleoplasm, as was previously reported [29]. Similarly, in the same report, U1-70K fused to GFP was shown to incorporate properly into snRNPs, suggesting that U1-70K fusions are functional. The number of speckles varied between cells averaging approximately 15–20 per cell. This was consistent with a previous report where U1-70K was shown to accumulate in speckles and in the nucleoplasm as well [29]. Since U1-70K is extensively phosphorylated in vitro and this phosphorylation affects its interaction with other SR proteins [18], [22], we asked if the subnuclear localization of U1-70K is affected by the phosphorylation status of the cell. For this analysis, we treated GFP-U1-70K expressing protoplasts with the kinase inhibitor, staurosporine, or the phosphatase inhibitor, okadaic acid, and studied changes in the pattern of subnuclear distribution of U1-70K. The most dramatic effect observed was with staurosporine treatment, which within two hours of application, caused redistribution of U1-70K from numerous smaller and round speckles on the background of diffused nucleoplasm in control cells to irregularly shaped larger speckles, which appeared coalesced throughout the 3-D space of the nucleus (Figure 1). Very little diffused distribution was left in staurosporine treated protoplasts. Okadaic acid, on the contrary, did not alter the localization pattern of U1-70K (Figure 1). Since splicing and transcription are coupled events [30], we investigated the effect of transcription inhibitors on the subnuclear distribution of U1-70K. Figure 1 shows that the pattern of subnuclear distribution in transcription-inhibited cells is similar to controls indicating that subnuclear distribution of U1-70K is not affected by the transcription status of the cells.

**Figure 2:**
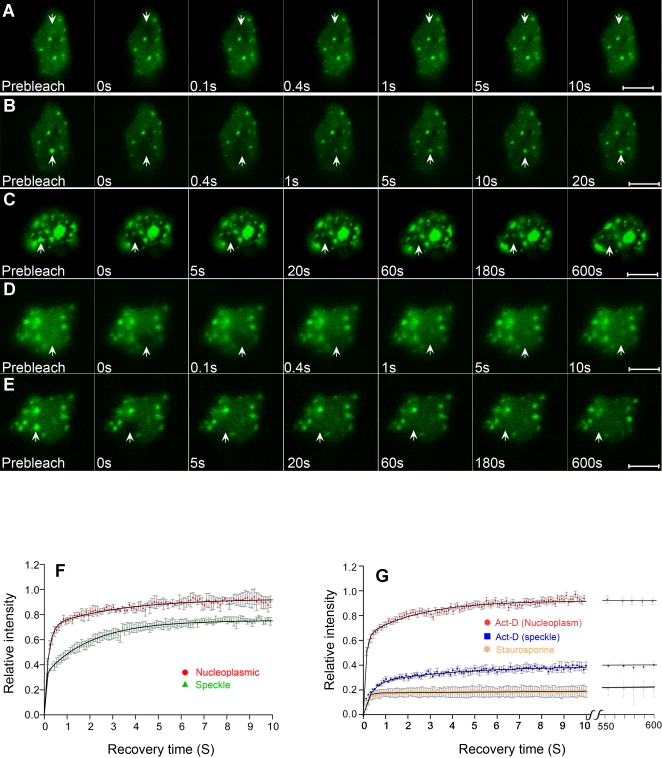
Mobility of GFP-U1-70K depends on transcription and protein phosphorylation. A nucleoplasmic area or a speckle indicated by an arrow was bleached with a high intensity 488 nm laser and photographed immediately and at regular intervals of ∼100 milliseconds after bleaching. Successive images taken after bleaching illustrate the level of return of fluorescence to bleached areas. (A and B) Fluorescence recovery after photobleaching (FRAP) images of control cells demonstrate that the GFP-U1-70K recovers fully within less than 10 seconds (A, nucleoplasm) or 20 seconds (B, speckle) indicating that U1-70K turns over very rapidly in speckles and nucleoplasm. (C) FRAP images of a representative protoplast treated with staurosporine show very little or no recovery. (D and E) FRAP images of a representative protoplast treated with actinomycin-D show a control level mobility in the nucleoplasm (D) and significantly reduced mobility in a speckle (E). Bar = 5 µm. (F) Quantification of FRAP data in speckles and the nucleoplasm reveals that U1-70K displays slower mobility in the speckles than in the nucleoplasm. Each data point is the average of 7 to 11 nuclei. Error bars are SEMs. (G) Quantification of FRAP data of U1-70K in nuclei treated with actinomycin-D or staurosporine demonstrates that the mobility of U1-70K is reduced by inhibition of transcription or phosphorylation. Each data point is the average of 5–10 nuclei. Error bars are SEMs.

Nuclear proteins, depending upon the strength and duration of their association with other nuclear components, may move in the nucleus with kinetics ranging from milliseconds to hours [31]. However, the mobility properties of U1-70K are not known. In order to determine the kinetic properties of U1-70K we performed FRAP analyses on speckles and nucleoplasmic regions of the nucleus. Figures 2A and B, respectively, show a nucleoplasmic region and a speckle at pre- and post-bleach stages of a FRAP experiment. These pictures illustrate that U1-70K moves in the nucleus very rapidly on a millisecond scale. Quantification of these recovery rates averaged over several nuclei are presented in Figure 2F. In the nucleoplasm, the effective diffusion co-efficient (D_eff_), which is a measure of the apparent mobility of a protein, was 0.80 µm^2^ s^−1^, whereas in the speckle it was 0.31 µm^2^ s^−1^. Also, the immobile fraction in the nucleoplasm was 10% as compared to 26% in the speckles (Table 1). Together these observations suggest that U1-70K exhibits high mobility in the nucleus and that the mobility in the speckles is slower than in the nucleoplasm. Using a similar protoplast expression system, we have previously shown that GFP alone had a D value of approximately 15 µm^2^ s^−1^. Since GFP is not known to interact with plant proteins, its diffusion reflects free diffusion [32]. Based on the experimental D value of GFP and the relationship that diffusion co-efficient is inversely proportional to the cubic root of molecular mass (DαM^−1/3^) [33], the expected free diffusion of U1-70K-GFP in the absence of any interaction with other proteins, would be approximately 10 µm^2^ s^−1^. However, the experimentally-determined D_eff_ of U1-70K is approximately 12 (in nucleoplasm), and 31 (in speckles) times smaller than the expected D value of uncomplexed protein suggesting that U1-70K interacts with other nuclear components or is in a larger complex. We then investigated if the mobility of U1-70K was dependent upon the phosphorylation and transcription status of the cell. Protoplasts expressing U1-70K were treated with the phosphorylation inhibitor staurosporine for two hours prior to analyzing the mobility of U1-70K using FRAP. As shown in Figure 2C, the mobility of U1-70K in speckles was significantly reduced by staurosporine. Quantification of the mobility showed that approximately 81% of U1-70K was rendered immobile by staurosporine treatment (Table 1, Figure 2G). Similarly, D_eff_ was also reduced by staurosporine by 8-fold (Table 1). In the speckles, the mobility of U1-70K was significantly reduced by actinomycin-D, whereas in the nucleoplasm its mobility was similar to control (Figure 2D and 2E). Treatment with actinomycin-D reduced both the mobile fraction and the D_eff_ by approximately two-fold (Figure 2G, Table 1). These results indicate that the mobility of U1-70K is dependent upon the phosphorylation and transcription status of the cells.

**Table 1:** Diffusion coefficients and mobile fractions of U1-70K.

	D_eff_ (µm^2^ s^−1^)	% mobile
Nucleoplasmic	0.80±0.04	90±4.3
Speckle	0.31±0.01	74±2.10
Staurosporine	0.04±0.03	19±2.7
Actinomycin-D	0.18±0.17	38±5.5

D_eff_ = Effective diffusion (retarded diffusion resulting from binding); Values given are the means±SEM of at least 7–10 nuclei in at least two different experiments.

### Nuclear and speckle targeting domains in the Arabidopsis U1-70K protein

**Figure 3:**
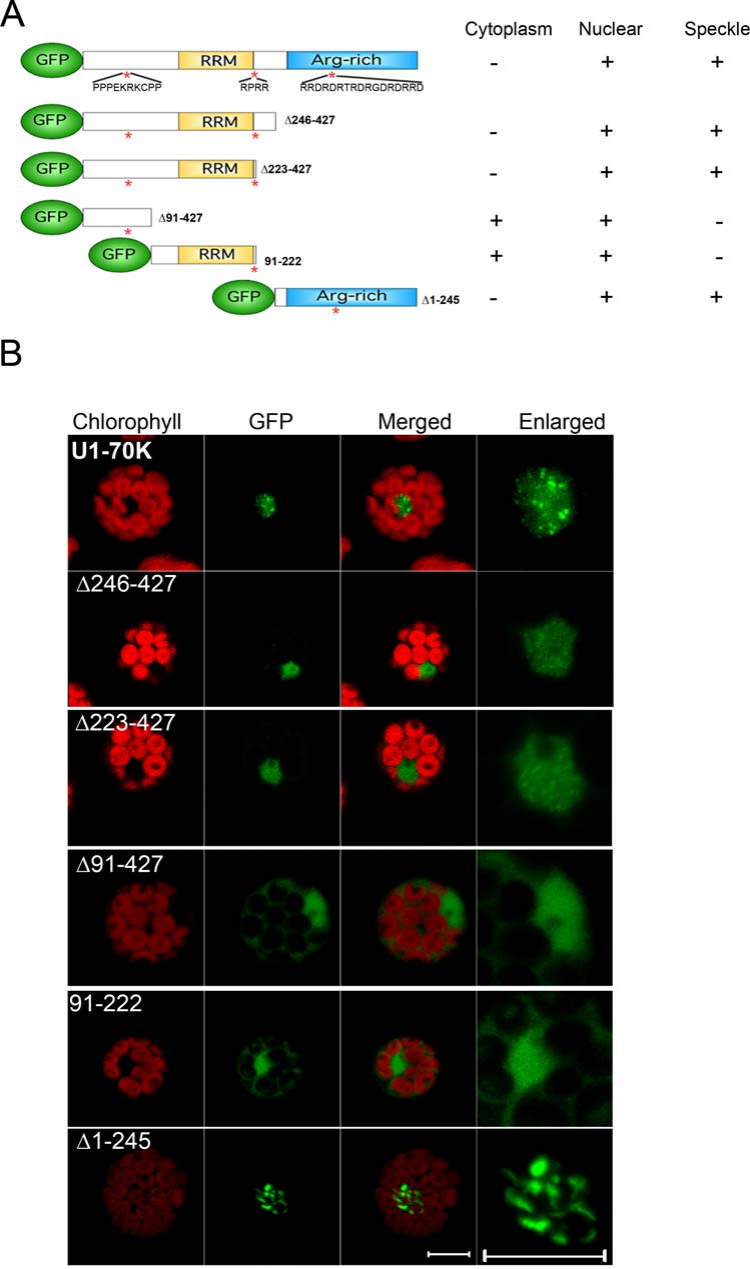
Speckle targeting signals in U1-70K are located in two different domains. (A) Constructs of full-length and deletion mutants of U1-70K tagged with GFP. Numbers after “Δ” are the deleted amino acids in U1-70K. Stars indicate the location of putative nuclear localization signals (NLS; amino acid sequences are shown below the stars). Table on the right shows the subcellular and subnuclear localization of the U1-70K deletion mutants. (+, present; −, absent). RRM, RNA recognition motif; Arg-rich, arginine-rich region; (B) Confocal images of Arabidopsis protoplasts expressing full-length U1-70K or a deletion mutant as indicated on each panel. Images in the right most column show a zoomed-in nuclear region of images shown under the GFP column. These results show that U1-70K has two independent nuclear- and speckle-targeting signals located in the N-terminal region up to the RRM domain and in the C-terminal arginine-rich region. Bars = 5 µm.

U1-70K has several domains, consisting of several predicted nuclear localization signals (NLS), a middle canonical RRM, an N-terminal region located before the RRM with no identifiable homology to known domains and a C-terminal serine-arginine rich region located after the RRM (Figure 3A). To identify the domains required for nuclear and speckle targeting, we constructed a series of GFP-tagged deletion constructs comprising different regions of U1-70K (Figure 3A). Full-length U1-70K localized exclusively to the nucleus with no detectable fluorescence in the cytoplasm or other organelles (Figure 1 and 3B). Deletion constructs consisting of the N-terminal half of the protein including RRM (amino acids 1–245, construct Δ246–427), and the C-terminal serine-arginine rich half (amino acids 246–427, construct Δ1–245) localized exclusively to the nucleus (Figure 3B). Interestingly, dividing the N-terminal half further into two halves, one consisting of amino acids 1–90 (construct Δ91–427) and the other one consisting of amino acids 91–222 (construct 91–222), abolished the exclusive nuclear localization (Figure 3B). Instead these deletion constructs localized to both nuclei and cytoplasm. Since there are no known predicted NLS at the point of truncation, these observations indicate that for nuclear localization, signals in both these regions are essential. Next, we examined the speckle targeting domain(s) in the U1-70K deletion constructs. As is shown in Figure 3B full length U1-70K localized mostly to speckles with a diffused nucleoplasmic fluorescence. The deletion constructs consisting of the N-terminal half (construct Δ246–427) or C-terminal half (construct Δ1–245) showed fluorescence in nuclear speckles/regions albeit with different patterns than the full length protein. In contrast to full-length U1-70K, the N-terminal half showed a mostly diffused nucleoplasmic pattern, with less obvious and small fluorescent speckles (Figure 3B). Dividing this deletion construct further into two halves, completely abolished the speckled pattern (Figure 3B, constructs Δ91–427 and 91–222). The C-terminal half displayed a very distinct localization pattern consisting of larger irregular shaped regions spread throughout the nucleoplasm with very little diffused distribution (construct Δ1–245, Figure 3B). Together, these observations show that at least two independent nuclear localization signals and speckle targeting signals are present in U1-70K and that proper speckle targeting/retention requires full-length U1-70K.

### Mapping of SR45 domains interacting with U1-70K

**Figure 4:**
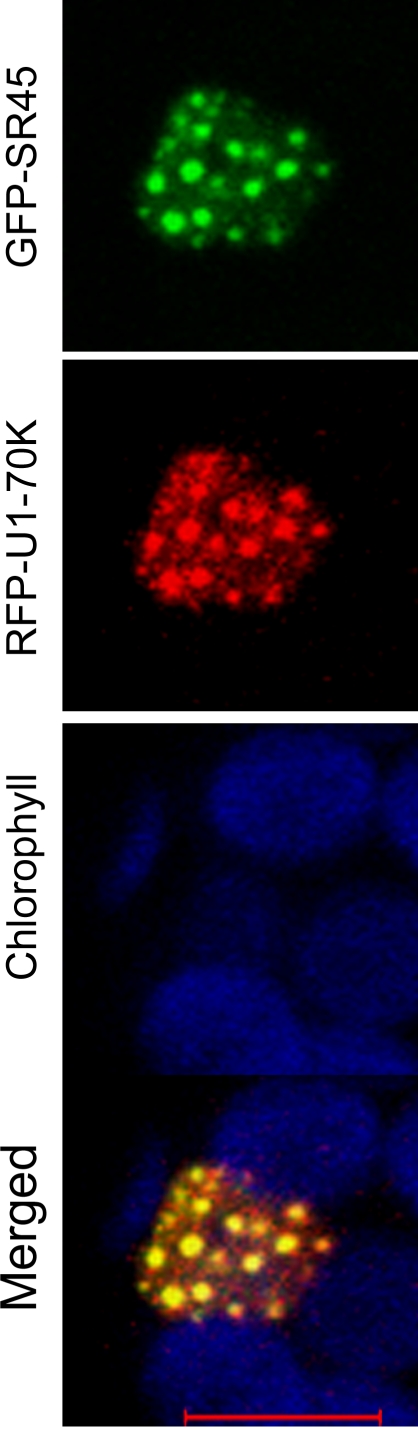
Co-localization of the Arabidopsis U1-70K and SR45. Confocal images of an Arabidopsis protoplast expressing GFP-SR45 fusion and RFP-U1-70K fusion. The merged image reveals extensive co-localization of these proteins. Chlorophyll autofluorescence is shown in blue. Bars = 5 µm.

**Figure 5:**
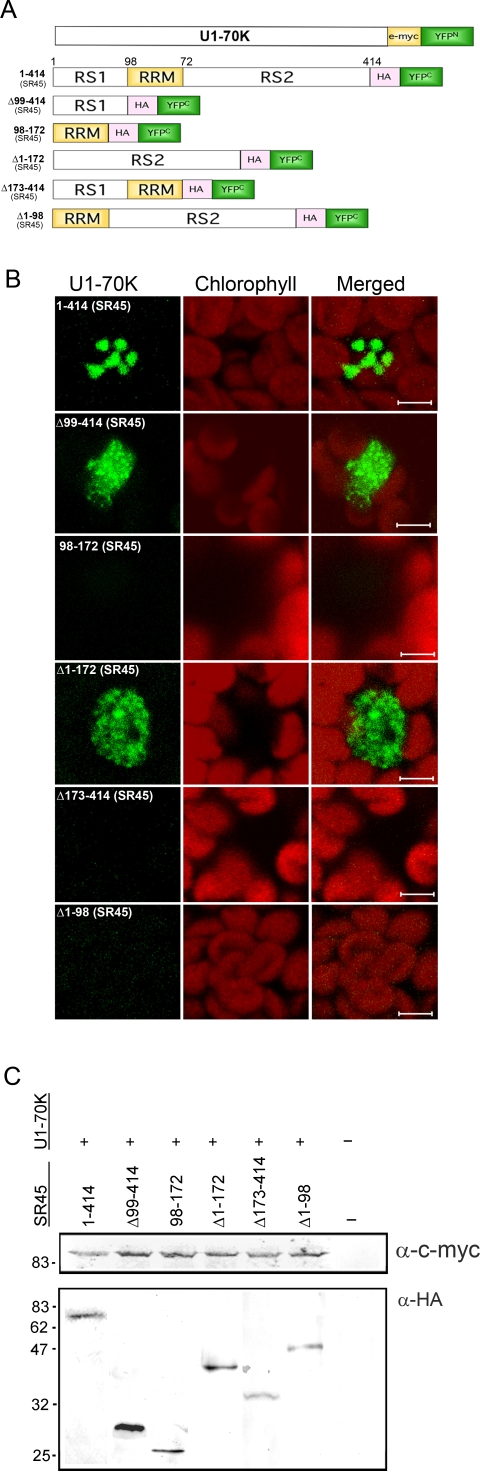
Bimolecular fluorescence complementation (BiFC)-based mapping of SR45 domains interacting with U1-70K. (A) BiFC vectors harboring U1-70K and a series of deletion mutants of SR45 were made by fusing full-length U1-70K to the N-terminal YFP fragment, YFP^N^ (amino acids 1–155) and of SR45 full-length and deletion mutants to the C-terminal YFP fragment YFP^C^ (amino acids 156–239) as described in Materials and Methods. Numbers after “Δ” show the deleted SR45 amino acids. C-myc, c-myc peptide; HA, hemagglutinin peptide. RS1 and RS2, arginine/serine-rich domain 1 and 2; RRM, RNA recognition motif. (B) BiFC images of Arabidopsis protoplasts co-transfected with U1-70K-YFP^N^ and a full-length or deletion mutant of SR45-YFP^C^ as indicated on each panel show that SR45 interacts with U1-70K through its RS1 or RS2 domain. Bar = 5 µm. (C) Western blot analyses show protein expression levels of U1-70K-YFP^N^ and of full-length and deletion mutants of SR45 fused to YFP^C^. The U1-70K-YFP^N^ was detected with an anti-c-myc-HRP (upper panel) and SR45 and its deletion mutants fused to YFP^C^ were detected with an anti-HA-HRP antibody (lower panel).

Although U1-70K interacts with several SR proteins in vitro, so far no studies have reported their in vivo interactions. Here we showed that U1-70K co-localizes with SR45 in the nucleus (Figure 4) suggesting that they may be interacting. To address this question, we employed the Bimolecular Fluorescence Complementation (BiFC) approach, which is based on the reconstitution of YFP fluorescence when the two split halves of YFP linked separately to two putative interacting proteins are brought together in close proximity only when the two tagged proteins interact [26]. This technique has been employed for studying in vivo protein-protein interactions in different cellular compartments and organisms including plants [34]. The Arabidopsis SR45 has a well-defined modular structure consisting of an N-terminal RS1, a middle RRM and a C-terminal RS2 domain. We, therefore, tested which domains of SR45 are responsible for interacting with U1-70K. U1-70K was fused to YFP^N^ and the domain deletion constructs of SR45 were fused to YFP^C^ (Figure 5A). Previously, we have shown that phenotypic and splicing defects observed in an *sr45* knockout mutant were rescued by SR45 fused to GFP [24], indicating that SR45-YFP^C^ fusions used in this study are functional. U1-70K-YFP^N^ was co-transfected with each of the SR45-YFP^C^ deletion constructs into protoplasts and observed for the reconstitution of YFP fluorescence. As shown in Figure 5B, YFP fluorescence was observed with full-length SR45 and U1-70K mostly in the speckles. Examination of the deletion constructs revealed that a combination of U1-70K with the RS1 or RS2 domains of SR45 also showed fluorescence, suggesting that these two domains interact with U1-70K independently. The pattern of fluorescence of these two constructs was however very different from the full length SR45. Instead of fewer and larger speckles with full-length SR45 protein, there were numerous smaller speckles with the RS1 or RS2 domains spread all over the nucleus (constructs Δ99–414 and Δ1–172, Figure 5B), which resembled the pattern of full-length U1-70K (Figure 1). In addition, with these deletion constructs, a substantially higher diffused fluorescence was also evident throughout the nucleus (Figure 5B). The RRM domain alone did not show any reconstitution of YFP fluorescence indicating that RRM does not associate directly with U1-70K (construct 98–172, Figure 5B). Interestingly, constructs that contained RRM with RS1 or RS2 domains (i.e. Δ173–414 and Δ1–98, Figure 5B), completely abolished the reconstitution of YFP fluorescence observed with RS1 or RS2 alone, indicating that RRM plays a key role in regulating the interaction of SR45 with U1-70K. To make sure that the absence of interactions with these constructs was not due to a lack of their protein expression, we performed Western blot analyses with protein samples isolated from protoplasts co-transfected with these constructs and U1-70K-YFP^N^. As shown in Figure 5C, all constructs that did not reveal any interaction with U1-70K, expressed proteins and the amount of protein is comparable to the constructs that did show interactions. From these results, we conclude that the interaction of SR45 with U1-70K is mediated by the RS1 or RS2 domain and that the presence of both RS1 and RS2 domains with RRM is necessary for interaction with U1-70K.

### The subnuclear distribution of U1-70K/SR45 complex is affected by protein phosphorylation

**Figure 6:**
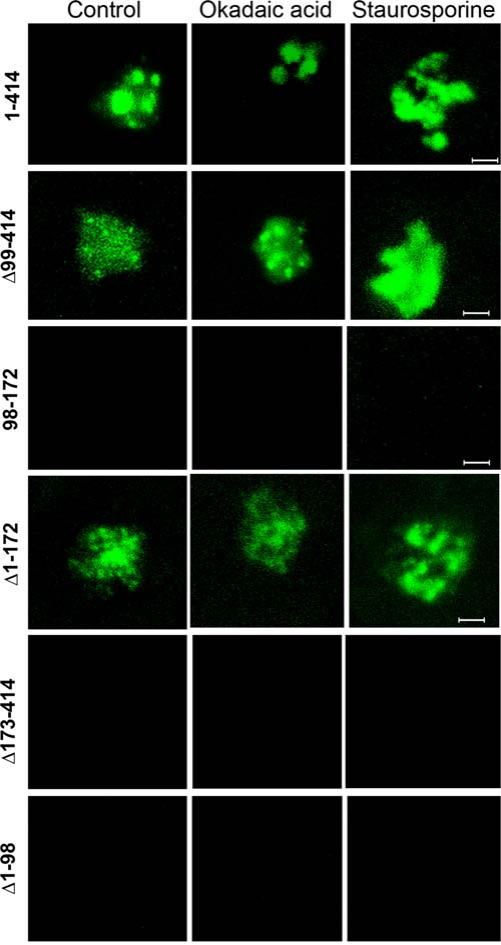
Effect of phosphorylation and transcription on the subnuclear localization pattern of SR45/U1-70K BiFC complexes. Arabidopsis protoplasts co-expressing a U1-70K-YFP^N^ and an SR45 full length and deletion mutants as indicated on each panel were treated with staurosporine or okadaic acid. Images show staurosporine re-organized the SR45/U1-70K BiFC complexes into irregular shaped speckles. Bar = 5 µm.

The interaction between some spliceosomal proteins is regulated by phosphorylation/dephosphorylation. Further, inhibition of phosphorylation and transcription re-localized SR45 to irregularly shaped speckles [25], [32]. Here we tested if the interaction between U1-70K and SR45 is regulated by phosphorylation and if phosphorylation inhibits U1-70K/SR45 complex localization pattern. Within two hours of inhibiting kinase activity by staurosporine, the SR45/U1-70K was reorganized into irregularly shaped enlarged speckles indicating that the subcellular localization of this complex is regulated by phosphorylation (Figure 6). Although the BiFC interaction is thought to be irreversible [reviewed in 34], there are several studies indicating that the BiFC complex could be dissociated in a time-course dependent manner or when association between the interacting proteins is disrupted by drugs [35]–[37]. This suggests, that in contrast to previous beliefs, the BiFC complex can be dissociated at least in some cases under in vivo conditions. Considering these studies, we reasoned that if the regulation of interaction between U1-70K and SR45 by (de)phosphorylation is strong enough to overcome the irreversibility of BiFC interaction, then we should see a change in the fluorescence when phosphorylation is altered by inhibitors. To test this hypothesis, we examined any loss of fluorescence, as would be expected if changes in phosphorylation would disrupt the interaction between SR45 and U1-70K, by extending our observations to six hours. Still no loss in fluorescence was detected, indicating that the dissociation of U1-70K/SR45 complex is probably not affected by protein phosphorylation. However, because of the reported irreversible nature of the BiFC interaction in some cases, further studies are needed to substantiate this conclusion. To test if inhibition of phosphorylation would prevent the association of SR45 with U1-70K, we treated protoplasts with staurosporine right after transfection of protoplasts. First, staurosporine treatment did not prevent the reconstitution of YFP fluorescence (data not shown), suggesting that inhibition of protein phosphorylation does not prevent association of SR45 with U1-70K. Second, instead of rounded speckles as seen in control, in the staurosporine-treated protoplast, we observed irregularly shaped enlarged speckles, confirming our observation that inhibition of protein phosphorylation causes accumulation of the SR45/U1-70K complex into enlarged irregular shaped speckles. We also carried out similar analyses with all combinations of SR45 deletion constructs and U1-70K. Treatments with staurosporine, either right after transfection or after the appearance of YFP fluorescence, did not abolish the RS1 (Δ99–414)/U1-70K or the RS2 (Δ1–172)/U1-70K interaction. Similarly, none of the rest of the SR45 deletions/U1-70K combinations reconstituted any YFP fluorescence in response to inhibition of protein phosphorylation, suggesting that their interactions are not affected by phosphorylation (Figure 6). Similarly, okadaic acid treatment had no effect on the dissociation of SR45/U1-70K complex or any other combination of the deletion mutants of SR45 and U1-70K (Figure 6). These data suggest that the association and dissociation of SR45 with U1-70K is probably not affected by the phosphorylation status of the cells.

### The mobility of SR45/U1-70K complex is slower than either protein alone

**Figure 7:**
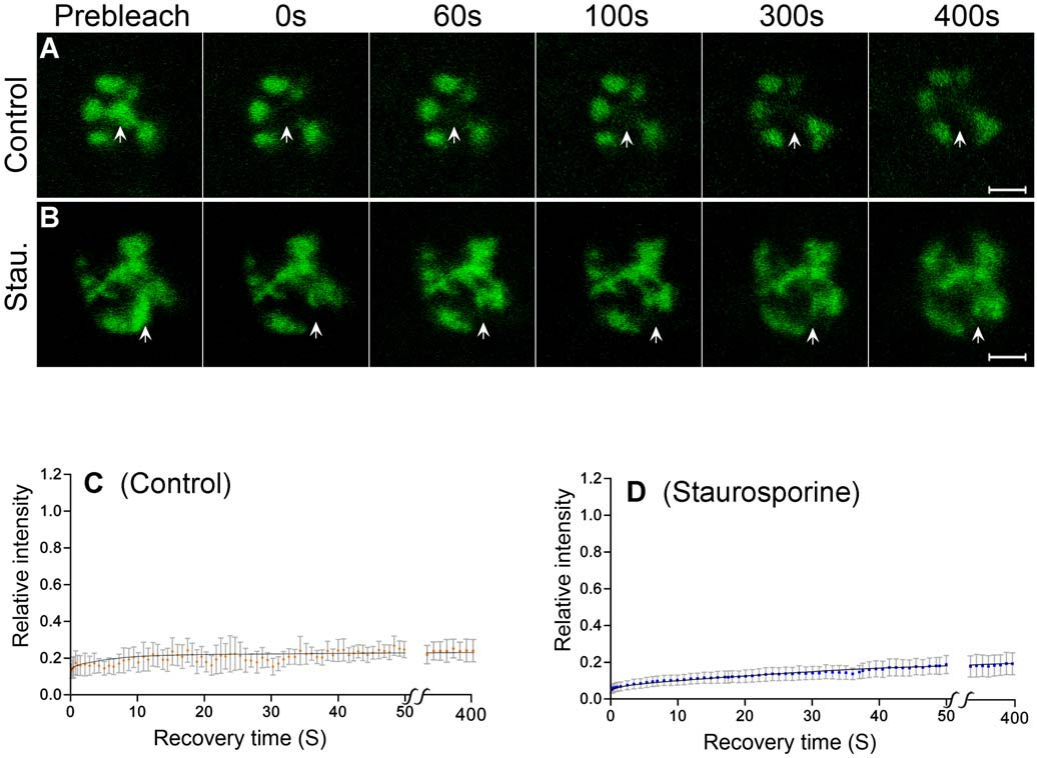
FRAP analyses of SR45/U1-70K BiFC complex. (A and B) An area indicated by an arrow in a protoplast co-expressing SR45-YFP^C^ and U1-70K-YFP^N^ was bleached with a high intensity 488 laser and imaged at regular intervals for up to 400 seconds. Shown are images immediately before bleach (pre-bleach) and at selected time-points after bleaching. Control is an untreated protoplast; stau. is a protoplast treated with staurosporine for two hours. Bar = 5 µm. (C and D) Quantification of FRAP of SR45/U1-70K BiFC complexes in protoplasts. The data suggest that SR45/U1-70K complex moves very slowly in the cells and this movement is not affected by phosphorylation. For discussion, see text. Each data point is the average of 7 nuclei. Error bars are SEMs.

The kinetic properties of SR45 have been reported previously [32], whereas that of U1-70K were determined in this report. These analyses, however, do not distinguish between the kinetic properties of these proteins when alone and in complex with other proteins. The reconstituted YFP fluorescence results from the interaction of U1-70K and SR45, thus providing us with a tool to investigate the kinetics of the U1-70K/SR45 complex with FRAP. As shown in Figure 7, FRAP analyses of the BiFC of SR45 and U1-70K show no or very little recovery over a period of at least 10 minutes. These observations are in contrast to the mobility of U1-70K alone (Figure 3) or SR45 alone [32], indicating that the SR45/U1-70K complex remains in the speckles longer than SR45 or U1-70K alone . To assay if inhibition of phosphorylation would change the kinetics of SR45/U1-70K complex, we also carried out FRAP analyses on protoplasts treated with staurosporine. A subtle reduction in the recovery kinetics of SR45/U1-70K complex was observed with staurosporine (Figure 7). Since our experimental set up does not allow us observations longer than a few minutes, we cannot rule out the possibility of a stronger effect, if any, over longer durations. Nevertheless, our data show that these inhibitors do not accelerate the kinetics of SR45/U1-70K complexes.

## Discussion

In vivo kinetic analyses with several nuclear proteins have provided convincing evidence that a majority of them exhibit high mobility and are dynamically associated with high order complexes exchanging continually between different sub-nuclear domains [31]. Since U1-70K has important functions in the early steps of spliceosomal assembly by defining the 5′ splicing site through interaction with U1 snRNA and several other U1 snRNP and non-snRNP proteins, it is important to study its biophysical properties and the dynamics of its interaction with other splicing-related proteins. In this report, we first investigated the subcellular localization and kinetic properties of U1-70K and then using a combination of BiFC with FRAP and several pharmacological inhibitors, we studied the interaction and the dynamics of that interaction with a plant-specific SR splicing factor, SR45.

### U1-70K is a highly mobile nuclear protein and its mobility is dependent upon phosphorylation and transcription

U1-70K is an important component of U1 snRNP and its interaction with SR proteins and other components is critical for the pre-spliceosomal assembly. Yet, it is not known if U1-70K is bound to its targets statically or is dynamically associated with it. Here, for the first time, we report analyses addressing these issues. Our data show that U1-70K is a highly mobile protein moving on a millisecond-scale in the nucleus and that it is in constant flux between speckles and nucleoplasm. Based on estimation from our data it would take less than 50 seconds for U1-70K to traverse an average size nucleus of 10 µm diameter. This provides an efficient means of delivering U1-70K to the site of its action or back for recycling. These data fit well with the “stop-and-go” model recently proposed for the function of several nuclear proteins [31]. According to this model, a nuclear protein scans the nuclear space for its interacting partners and is slowed down due to this interaction with a less mobile nuclear component. Our data do not allow us to precisely reveal the type and size of the U1-70K complex. Nevertheless, the slower mobility of U1-70K than would be expected for a protein of similar size if it were freely diffusing shows that U1-70K is part of a higher order complex. Such a high order complex could be U1 snRNP either in transit to or from the site of splicing and/or engaged in the process of splicing. The slower mobility of U1-70K in the speckles than in the nucleoplasm also suggests that in the speckles it interacts with a lesser mobile component perhaps pre-mRNA still attached to the chromatin at the sites of transcription. This interpretation draws support from the observations that splicing and transcription take place concurrently and that sites of transcription, as determined by br-UTP incorporation or RNA-FISH, usually colocalize with or localize to the periphery of splicing factor speckles [e.g. 38], [39].

In metazoan systems, completion of splicing requires a cycle of phosphorylation-dephosphorylation at different stages of splicing. For example, phosphorylation of ASF/SF2 increases its interaction with U1-70K and is essential for the assembly of spliceosomes [17], [40], whereas inhibition of protein dephosphorylation prevents completion of splicing [23], [41], [42]. Dephosphorylation of U1-70K is essential for the resolution of the spliceosome but not for the assembly of the spliceosome [23]. A comparison of the mobility and subcellular localization of U1-70K under conditions when phosphorylation was modulated shows that the phosphorylation status of the cell strongly influences the spatial distribution and kinetics of U1-70K. Similarly, inhibition of transcription would also consequently lead to a halt in splicing and mobility of U1-70K. Morphological analyses with several SR splicing factors have demonstrated that they rearrange into larger misshapen speckles after inhibition of phosphorylation [25], [32], [43], [44]. It was interesting to see that in a similar manner U1-70K also rearranged into enlarged speckles, suggesting that the phosphorylation-dependent subnuclear localization may be a general feature of splicing-related proteins.

### U1-70K has at least two independent nuclear and speckle targeting signals

The Arabidopsis U1-70K has two independent nuclear localization signals (NLS) located in the N-terminal and C-terminal half of the protein. One of the NLSs in human U1-70K was mapped to the RRM domains [45]. Notably, however, in our analyses, the RRM domain of Arabidopsis U1-70K alone was not sufficient for exclusive localization to the nucleus. Comparison of the amino acid sequences of the NLS in human U1-70K RRM (DGKKIDGRR) with the Arabidopsis U1-70K sequence (DGKQIDGRR) reveals a single amino acid change of K to Q. Since changing the KK dipeptide (charged) to FP (uncharged) in human U1-70K abolished its nuclear localization, it may be that a K to Q change in amino acid-201 present in the Arabidopsis U1-70K may have resulted in the loss of nuclear localization of RRM of U1-70K. Instead, the Arabidopsis U1-70K, in addition to the RRM requires the N-terminal unique region upstream of the RRM for exclusive nuclear localization. These data suggest that the NLSs in U1-70K may consist of multiple regions. The unique N-terminal region and the RRM of U1-70K contain two putative NLSs at amino acids 64–73 and 200–208, respectively, which together may behave in an additive fashion in targeting U1-70K to the nucleus. The C-terminal half of the Arabidopsis U1-70K contains a putative NLS located at amino acids 315–331, which is rich in basic amino acids and is very likely the second NLS in U1-70K.

Analyses with U1-70K deletion mutants allowed us to identify two different regions as the speckle localization signals: the first 222 amino acids including the RRM, and amino acids 246–427, primarily comprising the arginine-rich C-terminal region. Speckle targeting signals for several splicing factors have been localized to RS domains [32], [44], [46] or to the RRM regions [46], [47]. Although U1-70K lacks a typical RS domain and the proposed speckle targeting signals consisting of three to four basic amino acids followed by an RS dipeptide [48], its C-terminal region is rich in basic arginine residues and is considered equivalent to a typical RS domain found in SR splicing factors. Because of this functional similarity, the arginine-rich domain of human U1-70K has been shown to be sufficient for interacting with ASF/SF2 in vitro. Similarly, the arginine-rich domain of plant U1-70K also interacts with several SR proteins [20], [21]. Considering these facts, it is possible that the speckle targeting behavior of the arginine-rich region of U1-70K is because of its interaction with other speckle localized proteins. Alternatively, its speckle-targeting features might constitute a novel speckle-targeting domain. Similarly, the RRM alone was also not sufficient for speckle targeting. Instead both RRM and the N-terminal region were essential for speckle targeting. The uncharacteristic speckle targeting feature of the N-terminal and C-terminal halves of U1-70K, which were very distinct from full-length protein strongly suggests that for proper speckle targeting and perhaps dynamic targeting to subnuclear locations other domains of U1-70K are also essential.

### The Interaction of U1-70K with SR45 is mediated by the RS1 or RS2 domain of SR45

Among the SR proteins in plants, SR45 is unique in that it has two RS domains, one at the N-terminus and the other at the C-terminus. Considering the presence of two RS domains in SR45, our objective here was to determine which one of these two RS domains associates with U1-70K. Our results show that both RS domains associated with U1-70K independently. The pattern of localization of these interactions was, however, different from full-length SR45. Instead of localizing to enlarged speckles, we observed a pattern that resembled more like that of U1-70K alone. These observations suggest that RS1 or RS2 associates independently with U1-70K in regions wherever U1-70K is present. More interestingly the presence of the RRM with either of the RS domains completely eliminated these associations. Considering the domain organization, the SR45 deletion construct consisting of RRM+RS2 is comparable to several SR proteins that contains a single N-terminal RRM and a C-terminal RS domain, such as SR33, which has been shown previously to interact with U1-70K [1], [21]. Based on these observations, an interaction between this deletion construct and U1-70K was conceptually expected. On the contrary, however, we did not observe any association between U1-70K and the SR45 deletion construct consisting of RRM+RS2. Similarly, no association was observed between U1-70K and RS1+RRM. A possible explanation for these observations is that the presence of RRM in these deletion mutants, might sterically hinder their interaction with U1-70K. Together these data suggest that SR45 has evolved with unique domain organization and that when bound to RNA it requires the presence of both RS domains for interaction with U1-70K. Given the fact that a serine-arginine rich domain in U1-70K is sufficient and necessary for binding to the RS domain of SR proteins [18], [20], [21], the interaction of both RS domains of SR45 independently raises an important question of how these two domains would interact with the same site. It is likely that each RS domain interacts with U1-70K differentially at different stages of splicing and may be important during the rearrangement of spliceosomes during splicing. Conversely, since SR45 is also known to interact with SR33, it is also possible that one RS domain binds to U1-70K whereas the other binds to another SR protein during the assembly of the spliceosome.

### The U1-70K/SR45 BiFC complex exhibits slower mobility than either SR45 or U1-70K alone

So far FRAP analyses have been primarily employed for studying the kinetics of individual proteins tagged to a fluorescent protein [32], [43], [49]. This approach, however, does not resolve whether the determined kinetics for a protein reflects a situation when it is alone or in complex with other components. We reasoned that performing FRAP analyses on the SR45/U1-70K BiFC complexes will reveal the kinetics of this complex. The SR45/U1-70K complexes exhibit very slow mobility, indicating that SR45/U1-70K complexes reside in the speckles for a longer time probably in a storage form consistent with the proposed role of speckles as storage sites for pre-mRNA processing factors [28]. Using a similar BiFC approach with two RNA binding proteins, Y14 and NXF1, it was shown that approximately half of the fluorescence recovered within seconds after photobleaching [50]. This observation suggests that not all BiFC complexes are immobile. Several studies have suggested that a cycle of phosphorylation-dephosphorylation of SR and other splicing factors is essential for splicing [17], [23], [40], [51]. The interaction of ASF/SF2 to U1-70K was enhanced by the phosphorylation of RS domain [17], but that to self, hTra2α and SRp40 was reduced whereas that to U2AF^35^ was not affected [17], [40], implying that the phosphorylation state of SR proteins determine the choice of interacting partner. Two observations in our analyses indicate that phosphorylation of SR45 and/or U1-70K may not be essential for their association or dissociation. First, inhibition of protein phosphorylation after the formation of U1-70K/SR45 did not result in the disruption of the complex although a clear rearrangement of the complex into enlarged irregular speckles was detected. Second, if the interaction between SR45/U1-70K is positively affected by phosphorylation, then the addition of staurosporine right after transfection of protoplasts before the formation of complexes should prevent the phosphorylation of SR45 and U1-70K and their interaction. Our results are not consistent with such a scenario as addition of staurosporine did not prevent the U1-70K/SR45 BiFC complex formation (data not shown). Since the BiFC complex is essentially irreversible in some cases, the occurrence of artifacts resulting from such behavior of BiFC complexes can not be ruled out altogether [reviewed in [34]. However, several studies have shown that the BiFC complexes under in vivo conditions are reversible [35]–[37]. For example BiFC was observed between IκBα and MEKK3, and IκBβ and MEKK2 within two minutes of stimulation with TNF-α elicitor [36]. These associations reversed within a time-frame (15 min for IκBα +MEKK3, and 60 minutes for IκBβ+MEKK2) consistent with findings obtained with biochemical analyses [36]. We speculate that the irreversibility of the BiFC complex depends on the relative strength of forces that keep the split YFP halves together versus the forces that drive two proteins to dissociate after a secondary modification such as phosphorylation. The greater the force that keeps split YFP halves together the higher will be the irreversibility and vice versa. In view of these reports, further studies are needed to verify the mobility properties observed here with the SR45/U1-70K BiFC complex. Using several phosphatase inhibitors and a dephosphorylation-resistant thiophosphorylated metazoan U1-70K, it was shown that phosphatase activity is essential for both catalytic steps of splicing but not for the assembly of U1 snRNP and spliceosomes [23], [41], [42]. In our analyses, inhibition of phosphatase activity did not disrupt the BiFC complex, suggesting that a phosphatases activity is probably not involved in the assembly of early spliceosomal complexes.

In summary, we have demonstrated that U1-70K roams the cell nucleus rapidly ensuring its availability throughout the nucleus for taking part in splicing. The observation that the mobility of U1-70K was stopped by the inhibition of transcription indicates that the mobility of proteins in the nucleus may be regulated by their need. Using a combination of FRAP and BiFC, we suggest that the mobility of the SR45/U1-70K complex is different from the individual proteins. Applying FRAP to BiFC complexes between other protein pairs involved in diverse cellular processes will have significant advantage over in vitro methods in understanding the functional organization and regulation of protein complexes on a system level in vivo.

## Materials and Methods

### Plasmid constructs and the Arabidopsis protoplast transfection assays

GFP-fused full length and truncated mutants of U1-70K were constructed as follows. Full length or truncated mutants as shown in Figure 3A were PCR-amplified using a full-length U1-70K cDNA cloned in pBS (KS+) as a template with forward and reverse primers containing *Sac*II and *Xma*I sites respectively. Sequences of these primers and their respective amplified U1-70K DNA fragments and their coding regions are provided in Table S1. Amplified PCR products were digested with *Sac*II and *Xma*I and cloned to the C-terminus of GFP in the *Sac*II/*Xma*I sites of pGFPII (GA5) plant expression vector resulting in pU1-70K-GFPII. The U1-70K-Red fluorescent protein (RFP) fusion was constructed by replacing the GFP in pU1-70K-GFPII with a PCR-amplified RFP fragment. PCR was performed with primers that contained *Xba*I on the forward and *Sac*II on the reverse primer. The template was pDsRed-Monomer (Clontech), which codes for a monomeric RFP. For Bimolecular Fluorescence Complementation (BiFC) analyses, a full-length U1-70K fragment except stop codon was PCR amplified with forward and reverse primers containing *Sal*I and *Xma*I sites, respectively, and cloned in *Sal*I/*Xma*I sites of pSPYNE-35S/pUC-SPYNE vector resulting in U1-70K-YFP^N^ vector. Similarly, full-length and truncated mutants of *SR45* were amplified with forward and reverse primers containing *Sal*I and *Xma*I sites, respectively, and cloned in the *Sal*I/*Xma*I sites of pSPYCE-35S/pUCSPYCE generating the SR45-YFP^C^ constructs (Figure 5A). Primer sequences and their respective DNA fragment and corresponding amino acid sequences are given in Table S1. All fusion constructs were verified by sequencing.

Transient expression in Arabidopsis mesophyll protoplasts were conducted using a standard polyethylene glycol (PEG) transfection protocol [52] Briefly, protoplasts were isolated from 4 week-old plants using 2.0% cellulase (Onozuka R-10) and 0.2% macerozyme (R-10, Yakult Hansha Co, Japan) in W5 medium (154 mM NaCl, 125 mM CaCl_2_, 5 mM KCl and 2 mM MES). Approximately 2×10^6^ protoplasts were transfected with 10 µg plasmid DNA with 40% PEG for 30 minutes. PEG was removed by diluting the protoplasts with W5 medium followed by centrifugation at 100× g for 2 minutes. The protoplast pellet was resuspended in W5 medium and incubated in 15-mL polypropylene conical tubes in the dark at 22°C for 18 hours. A 50-µL aliquot of the expressing protoplasts was transferred to a glass-bottom Petri dish fitted with a 30 to 70 µm thick cover slip and observed immediately under the microscope.

### Inhibition of phosphorylation, dephosphorylation and transcription

Transcription or protein phosphorylation was inhibited by treating protoplasts transfected with desired constructs with actinomycin D (CalBiochem) at 5 µg ml^−1^ or staurosporine (CalBiochem) at 10 µM for 2 hours. Similarly, protein phosphatase activity was inhibited by okadaic acid at 1 µM for 2 hours.

### Confocal microscopy and FRAP analyses

Confocal microscopy and FRAP analyses were conducted essentially as described [32] with a Zeiss LSM 510 Meta laser scanning microscope using a 63×, N.A. 1.4 oil-immersion apochromate objective. A fixed circular area of 1-µm radius or a speckle was bleached with 15 full-strength pulses of a 25 mW argon laser (488 nm). The bleaching routine started with four pre-bleach scans, followed by a bleaching scan that lasted for approximately 200 milliseconds. After bleaching, images were taken at the attenuated 0.1–0.5% laser transmission. The first image at the end of bleaching was taken immediately, the rest of the images were taken at a 98 to 250-millisecond constant interval for 60 seconds or until maximal recovery was reached. For quantitative FRAP analyses, fluorescence intensity of the bleached area and the entire nucleus was determined using LSM 510 software. Background-corrected intensities were normalized for photobleaching resulting from bleach-pulse and normal scanning according to the following equation: I = I_t_/I_0_×T_0_/T_t_, where I is the normalized intensity of the bleached area, T_0_, total nuclear intensity before bleach, T_t_, total nuclear intensity at time interval t after bleaching, I_0_, intensity of the bleached area before bleaching and I_t_, intensity of the bleached area at time intervals t after bleaching. The resulting normalized intensity data were fitted to the following exponential models: I = Y_max_(1−e^(−k*t)^), where Y_max_ is the final level of recovery, t is the time in seconds and k is recovery rate constant. All nonlinear curve fitting and the statistical comparisons were performed in GraphPad Prism 4.0 (http:/www.graphpad.com). Total maximal recovery values represent total mobile population. The immobile fraction was calculated as 1-mobile fraction, with 1 as 100% recovery. D_eff_ values were calculated according to the following formula: D_eff_  = 0.88x(r^2^/4xt_1/2_) [53], where, r is radius of the bleached area, t_1/2_ is the time when half of the final fluorescence has recovered. The predicted free diffusion co-efficient of U1-70K-GFP was determined as described previously [32], [33].

#### Bimolecular Fluorescence Complementation (BiFC) Assays

Bimolecular fluorescence complementation experiments were performed by co-transfecting U1-70K-YFP^N^ and SR45-YFP^C^ vectors or truncated mutants of SR45-YFP^C^ (Figure 5A) in the Arabidopsis mesophyll protoplasts as described above [26], [52]. Twelve to sixteen hours after transfection, reconstitution of YFP fluorescence was observed for up to 24 hours using a confocal microscope with the following YFP filter set up: excitation 514 nm, 458/514 dichroic, and emission 560-615 BP filter. FRAP of the BiFC complex was performed according to the set up exactly as described above.

#### Western blot analysis

Approximately 2×10^7^ leaf mesophyll protoplasts from 4-week old Arabidopsis plants were co-transfected with 50 µg of U1-70K-YFP^N^ and 50 µg full-length SR45-YFP^C^ vector or truncated SR45-YFP^C^ mutant vectors (Figure 5A) as described above [26], [52] and incubated for twelve to sixteen hours in dark. Protoplasts were collected by centrifugation at 300 g for 3 minutes, and resuspended in Laemmli buffer (constructs 1–414, Δ99–414, 98–172, Δ173–414) or in 100 mM phosphate-buffered saline, pH 7.5 supplemented with 6 M urea (Δ1–172 and ΔΔ1–98) and vortexed for 10 minutes at 4°C. Two volume of protein samples were mixed with one volume of 3× Laemmli buffer and boiled for five minutes. Protein samples were separated on duplicate 12% SDS-polyacrylamide gels and transblotted onto nitrocellulose membranes. Blots were blocked with 5% non-fat dry milk in Tris-buffered saline supplemented with 1% Tween-20 overnight at 4°C. One blot was probed with anti-HA-HRP (dilution 1∶400) and the other with anti-c-myc-HRP (dilution 1∶1000) (Roche) for 3 hours in blocking buffer. HRP activity was detected with 3, 3′-diaminobenzidine (DAB).

## Supporting Information

Table S1 Primers used for cloning the constructs used in this study.
(0.07 MB DOC)
