# Supplementary material for: Analyses of In Vivo Interaction and Mobility of Two Spliceosomal Proteins Using FRAP and BiFC
Source: PLoS One. 2008 Apr 16;3(4):e1953. doi: 10.1371/journal.pone.0001953 (PMC2278372; doi:10.1371/journal.pone.0001953)
Supplement: Table S1 — Primers used for cloning the constructs used in this study. (0.07 MB DOC) [file pone.0001953.s001.doc]

**Supporting Information.**

**Table S1**. Primers used for cloning the constructs used in this study

| Construct | 5’ Primer | 3’ Primer | Nucleotides | Amino acids |
| --- | --- | --- | --- | --- |
| U1-70K  full length | 5’-tcc**ccgcgg**catgggagactccggcgatcct-3’ | 5’-tccc**cccggg**acgaacatactctcgcgattc-3’ | 1-1281 | 1-427 |
| Δ246-427 (U1-70K) | 5’-tcc**ccgcgg**catgggagactccggcgatcct-3’ | 5’-tccc**cccggg**ttccccaacaatttcttcacc-3’ | 1-735 | 1-245 |
| Δ223-246(U1-70K) | 5’-tcc**ccgcgg**catgggagactccggcgatcct-3’ | 5’-tccc**cccggg**ccagtttgggacggttctacc-3’ | 1-666 | 1-222 |
| Δ91-247 (U1-70K) | 5’-tcc**ccgcgg**catgggagactccggcgatcct-3’ | 5’-tccc**cccggg**tggacgtattctggatcccc-3’ | 1-270 | 1-90 |
| 91-222(U1-70K) | 5’-tcc**ccgcgg**ccctaagccagaagttgaatta-3’ | 5’-tccc**cccggg**ccagtttgggacggttctacc-3’ | 273-666 | 91-222 |
| Δ1-245(U1-70K) | 5’-tcc**ccgcgg**ccaacaaccgcaagaagaacc-3’ | 5’-tccc**cccggg**acgaacatactctcgcgattc-3’ | 736-1281 | 246-427 |
| SR45 full length | 45BiFF  5’-acgc**gtcgac**atggcgaaaccaagtcgtggc-3’ | 45BiFR  5’-tccc**cccggg**agttttacgaggtggaggtgg-3’ | 1-1239 | 1-413 |
| Δ99-414 (SR45) | 45BiFF  5’-acgc**gtcgac**atggcgaaaccaagtcgtggc-3’ | 98BiFR  5’-tccc**cccggg**aagagattcttgaacagctttc-3’ | 1-294 | 1-98 |
| 98-172(SR45) | 98BiFF  5’-acgc**gtcgac**atgcttgttctccatgttgattct-3’ | 172BiFR  5’- tccc**cccggg**cgttgctttaacaacctttcc-3’ | 294-516 | 98-172 |
| Δ1-172(SR45) | 172BiFF  5’-acgc**gtcgac**atgctaccacctcgtcagaaagt-3’ | 45BiFR  5’-tccc**cccggg**agttttacgaggtggaggtgg-3’ | 519-1239 | 173-413 |
| Δ173-414(SR45) | 45BiFF  5’-acgc**gtcgac**atggcgaaaccaagtcgtggc-3’ | 172BiFR  5’- tccc**cccggg**cgttgctttaacaacctttcc-3’ | 1-516 | 1-172 |
| Δ1-98(SR45) | 98BiFF  5’-acgc**gtcgac**atgcttgttctccatgttgattct-3’ | 45BiFR  5’-tccc**cccggg**agttttacgaggtggaggtgg-3’ | 294-1239 | 98-413 |
| U1-70K  BiFC full length | 70KBiFF  5’-acgc**gtcgac**atgggagactccggcgatcct-3’ | 70KBiFR  5’- tccc**cccggg**acgaacatactctcgcgattc-3’ | 1-1281 | 1-427 |
| DsRed-M1 | DsRedF  5’-ctagtctagagtaccatggacaacaccgag-3’ | DsRedR  5’-ccgggcggtgaggccgagggtggcgcccct-3’ | 1-686 | 1-343 |
